# Multiparameter flow cytometry enables immune profiling and IFN pathway analysis in human minor salivary glands

**DOI:** 10.3389/fdmed.2025.1590516

**Published:** 2025-06-12

**Authors:** Eiko Yamada, Kalie Dominick, Rachel J. Kulchar, Joseph Twohig, Zohreh Khavandgar, Margaret Beach, Eileen Pelayo, Alan Baer, Paola Perez, Blake M. Warner

**Affiliations:** ^1^ Salivary Disorders Unit, National Institute of Dental and Craniofacial Research, National Institutes of Health, Bethesda, MD, United States; ^2^ NIDCR Sjögren’s Disease Clinic, National Institute of Dental and Craniofacial Research, National Institutes of Health, Bethesda, MD, United States

**Keywords:** salivary glands, Sjogren's disease, tissue dissociation, IFN pathway cytometry, flow cytometry

## Abstract

**Aim/Introduction:**

We aimed to achieve direct quantitative measurement of activated and therapeutically actionable pathways (e.g., Type-I interferon) in target organs of autoimmune disease using flow cytometry of human salivary glands. Sjögren's Disease (SjD) is a systemic autoimmune disorder characterized by lymphocytic inflammation and dysfunction of the lacrimal and salivary glands. Minor salivary glands are routinely biopsied and are used for the histopathological diagnosis of SjD. In this study, we optimized the dissociation, permeabilization, antibody panel, and analytical parameters to characterize both the immune and epithelial cells in the glands, and the activation status of a specified pathway by measuring intracellular phosphorylated proteins.

**Methods:**

Fresh human MSG biopsies were dissociated into single-cell suspensions and permeabilized under optimized conditions. MSG suspensions were stained for cell surface and intracellular markers then analyzed using nine-color conventional flow cytometry, including two intracellular markers.

**Results:**

Our optimized dissociation and permeabilization protocols for human MSG preserved key cell surface markers. Our flow cytometry panel identified major immune cell populations and distinguished epithelial cells via cytokeratin-18. We demonstrate the protocol's utility showing differential interferon pathway activity in SjD vs. healthy MSG leukocytes and epithelial cells. We provide guidance on panel selection, analytical capabilities, and the impact of cell yield on resolution using conventional flow cytometry.

**Conclusion:**

Our optimized protocol enables high-resolution characterization of immune and epithelial cell populations in human MSG, preserving key markers and capturing interferon pathway activity. Our protocol provides a robust framework for the direct study of immune heterogeneity and signaling dynamics in SjD at single cell resolution.

## Introduction

Sjögren's Disease (SjD) is a common autoimmune disorder characterized by chronic inflammation, progressive destruction, and dysfunction of the lacrimal and salivary glands, often accompanied by systemic manifestations (1). Despite extensive research efforts, no approved therapies effectively alter disease progression, largely due to its heterogeneity and the incomplete understanding of its pathogenesis. Diagnosis is based on a patient's clinical presentation, supported by the 2016 American College of Rheumatology/European League Against Rheumatism (ACR/EULAR) classification criteria, which include reduced salivary and lacrimal flow, corneal damage, anti-SSA autoantibodies, and lymphocytic sialadenitis with a focus score exceeding one per 4 mm^2^ (2). A substantial subset of SjD patients exhibit heightened Type-I interferon (IFN) activity in both the blood and salivary glands, suggesting a key role for this pathway in disease pathogenesis (3, 4). However, the precise cellular sources and upstream drivers of IFN signaling remain unclear. Characterizing immune and epithelial cell populations within affected glands using flow cytometry allows for direct assessment of IFN pathway activation, providing insights into pathogenic mechanisms and potentially identifying patients who may benefit from targeted IFN-blocking therapies.

Detailed characterization of immune subpopulations, including intracellular phospho-protein status, enables the identification of druggable pathways for targeted therapies. Traditional cell analysis methods are time-consuming and require larger tissue samples than standard clinical biopsies. Single-cell sequencing offers detailed transcriptional insights but has limited capacity for analyzing phosphorylated proteins (4–7). Mass spectrometry-based proteomics identifies thousands of proteins per cell but is costly and does not directly capture signaling pathway activation (8). Flow cytometry overcomes these limitations by enabling rapid, single-cell analysis of surface and intracellular proteins, modifications, and signaling pathways (9). Using fluorochrome-conjugated antibodies and other fluorescent reagents, this technique allows phenotypic characterization and validation of sequencing data (10). Recent advancements have expanded its capacity to analyze up to 50 markers simultaneously, enhancing its utility in immune profiling and drug development (11).

The aim of this study was to develop simple methodology for the analysis of minor salivary glands (MSG) using conventional and spectral flow cytometry. We developed this approach to characterize the composition of immune-infiltrated target organs in a common autoimmune disease, SjD, and directly measure the activation states of epithelial cells and immune cell subsets. As an illustrative example, we employed both canonical surface markers and intracellular proteins focusing on Type-I IFN pathway.

To ensure reproducible and specific results, we optimized enzymatic tissue dissociation and permeabilization methods to preserve cell surface markers, distinguish infiltrating immune cells from epithelial cells, and reliably detect intracellular proteins in whole MSG tissues. We also adjusted antibody and equipment settings to enhance detection sensitivity. Our conventional flow cytometry approach demonstrated consistent detection of epithelial and immune cells and revealed alterations in Type-I IFN pathway-related intracellular proteins in SjD glands compared to non-SjD controls. Specifically, our two seven-color conventional flow cytometry panels characterized up to five immune cell subsets, epithelial cells, and two Type-I IFN pathway-related intracellular proteins.

## Materials and methods

### Minor salivary gland sample collection

#### Subjects and ethical approval

Study participants provided informed consent prior to the initiation of any study procedure and were evaluated comprehensively, classified according to 2016 American College of Rheumatology (ACR) and the European League Against Rheumatism (EULAR) classification criteria (12). Comparator groups included subjects who did not meet 2016 ACR-EULAR criteria (non-SjD) or healthy volunteers (HV). All subjects were screened for evidence of systemic autoimmunity and received comprehensive oral, sialometric, rheumatological, and ophthalmological investigations. Clinical investigations were conducted in accordance with the Declaration of Helsinki principles. All studies using human samples were approved by the NIH IRB (15-D-0051, NCT00001390; 11-D-0172, NCT02327884).

#### Patient and public involvement

Neither patients, nor the public, were involved in the design, conduct, reporting, or dissemination plans of this research.

### Experimental design

**Figure 1:**
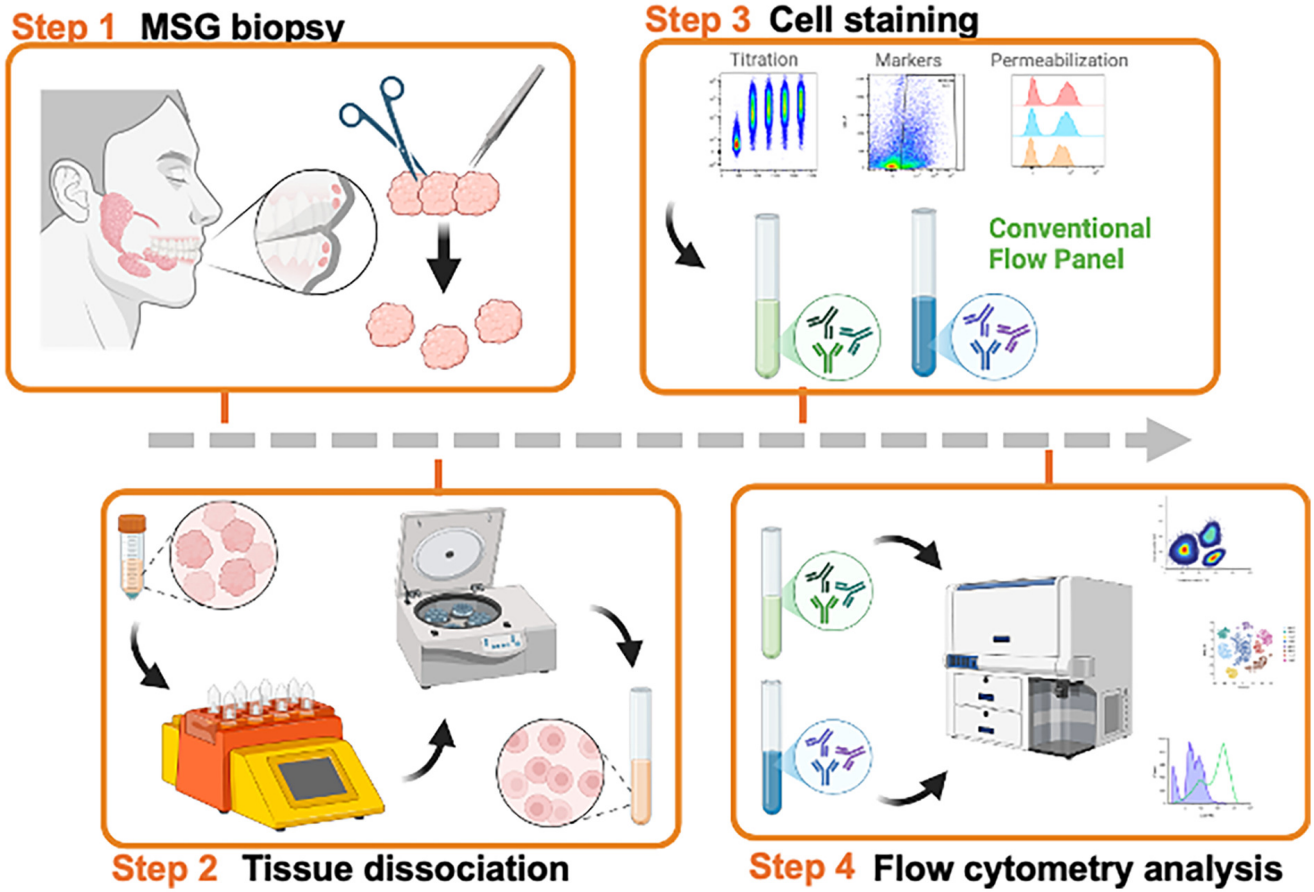
Graphic overview of our study. Created in BioRender. Warner, B. (2025) https://BioRender.com/wxk055a, Creative Commons Attribution License (CC BY).

A graphical overview of the methodology is shown in Figure 1. MSG biopsy tissues were dissociated to achieve single cell suspensions, permeabilized, stained with antibodies, and analyzed using conventional and spectral flow cytometers. The conventional flow cytometry panel differentiates total leucocytes (CD45+), pan-T cells (CD3+), T-helper (Th, CD4+) and cytotoxic T (Tc, CD8+) cells, B cells (CD19+), epithelial cells (KRT18+), and twoType-I IFN pathway intracellular markers, phosphorylated *p*-interferon regulatory factor 3 (IRF3) and *p*-nuclear factor-kappa B (NF-κB) (Supplementary Table S1).

### Tissue dissociation method for single cell

Fresh human MSG were processed for dissociation following modifications to previously reported methods (6, 13). Immediately after excision, MSG (2–3 glands per patient) were submerged in 5 ml of sterile, ice-cold RPMI and transported to the research laboratory for processing. Using a sterile disposable transfer pipette, MSGs were transferred to a sterile 100 mm tissue culture dish under a dissection microscope. The fibrous capsule of the glands was gently removed using ultra fine tapered-end stainless steel forceps; the individual gland lobules were separated and gently sectioned into ∼0.5 mm pieces using a #15 scalpel blade (*note: avoid excessive “mincing”*). Tissues were then suspended in 2.5 ml of DMEM (Gibco) and digested using Human Multi Tissue Dissociation Kit A (Miltenyi Biotec) or collagenase Ⅳ (Worthington) in an OctoMACS tissue disruptor (Miltenyi Biotec) using the multi A01 protocol with heated sleeves at 37°C. Single-cell suspensions were filtered through 70- and 30 μm filters. Filters were rinsed with 2 times the digestion volume with Hanks' buffered salt supplemented with 0.04% ultrapure, DNase/RNase-free, bovine serum albumin solution (Invitrogen**)** into same 15 ml low-binding sterile Falcon tube. Cells were centrifuged at 300 × *g* for 10 min at 4°C in a swing-arm bucket rotor and washed twice with same re-suspension solution. After the last centrifugation step, cells were suspended in 1 ml of resuspension solution and the number and viability were determined using a Trypan blue exclusion assay. Then, cells were fixed using 250 ml of BD Cytofix Fixation Buffer (BD) containing 4.2% formaldehyde for 2 h at 4°C and washed with staining buffer. For preliminary experiments, CD45 positive or negative cells in MSG were selected magnetically using MojoSort™ Human CD45 Selection Kit (BioLegend).

### Panel design approach

As part of a multi-modal study on immune cells, we analyzed key cell types relevant to both health and disease. To account for the complexity of known cell biomarkers and diverse cell functions, we combined basic phenotyping with targeted identification of specific populations, including markers of Type-I IFN pathway activation. These high-parameter panels enable detailed identification of cell subsets, characterization of infiltrating immune cells, and the generation of high-value data on key immune features.

To cross validate the frequencies of major immune cell compartments, five common lineage markers were included in the conventional panel to evaluate: total leukocytes (CD45+), pan T cells (CD3+), Th (CD4+) and Tc (CD8+) cells, and B cells (CD19+). This design ensured verification of immune cells infiltration and Type-I IFN activation performance. This validation is critical for identifying novel activities in samples with outlier behavior, ensuring these activities reflect novel biological phenomena rather than staining or data acquisition artifacts.

### Flow cytometry

#### Antibodies and titration

Ten fluorochrome-conjugated anti-human antibodies were used for the finalized panels (Table 1, Supplementary Table S1). Antibody titration was performed by staining healthy human peripheral blood mononuclear cells (PBMC) with four serial dilutions (Supplementary Figure S1A). Healthy PBMCs were used to titrate markers with positive signal that showed clear separation from background signal. To titrate the epithelial cell marker, the “salivary” model cell line, human salivary gland (HSG) cells (14–17), were used.

**Table 1:** Antibody list.

Marker type	Specificity	Fluorochrome	Clone	Purpose
Surface markers	CD45	BV711	HI30	Pan Leukocytes
	CD3	APC-Cy7	SK7	Pan T cell, NKT-Like cells
	CD4	PerCP-Cy5.5	SK3	CD4T and NKT-Like cells
	CD8	BV510	SK1	CD8T and NKT-Like cells
	CD19	BV605	HIB19	B cells
Intracellular markers	KRT18	DyLight 405	C-04	Epithelial cells
	pIRF3	PE	14G10A21	IFN Pathway activation
	pNF-kB	Alexa Fluor 488	D52C2	
	EpCam	BV421	9C4	Epithelial cells

#### Sample staining, instrumentation, and sample acquisition

Freshly biopsied human MSGs were dissociated as described above. Cells were stained with optimized concentrations of cell surface markers for 30 min at room temperature in the dark. After washing, cells were permeabilized with 100 ml BD Phosflow Perm Buffer II (64.9% methanol) for 30 min at −20°C, followed by another wash with staining buffer. Intracellular marker staining was performed overnight at 4°C in the dark. Reagents are listed in Supplementary Table S2.

Multicolor conventional flow cytometry quantified cell subsets and phosphorylation levels within gated populations. Data were acquired on a five-laser BD Symphony A3 cytometer and analyzed using FlowJo™ v10.8 (BD Life Sciences). Instrument setup and performance tracking were conducted daily using BD Cytometer Setup and Tracking (CS&T) beads. Experimental voltages were determined through internal optimization with fixed healthy PBMCs. The Symphony instrument was operated with BD FACSDiva software v9.1 (Supplementary Table S3).

### Fluorescence compensation

Compensation matrix generation on BD Symphony data was completed by creating single color controls using healthy PBMCs or HSG for KRT18. Single color controls were stained with the same protocol and titer as the experimental conditions. Five thousand events were recorded for compensation controls on the BD Symphony cytometer. Compensation matrices for conventional cytometer data were calculated in FlowJo (BD, Version 10.8.2).

### Data collection and statistical analysis

Flow Cytometry Standard (FCS) files from the BD Symphony conventional cytometer was exported in FCS 3.1 format. FCS files were manually gated to remove doublets, debris, and each cell subset using FlowJo (BD Biosciences, Version 10.8.2). OMIQ (Dotmatics) was used to visualize FCS files data as opt-SNE, an automated toolkit for t-SNE parameter selection (18).

## Results

### Selection and optimization of dissociation and permeabilization conditions

**Figure 2:**
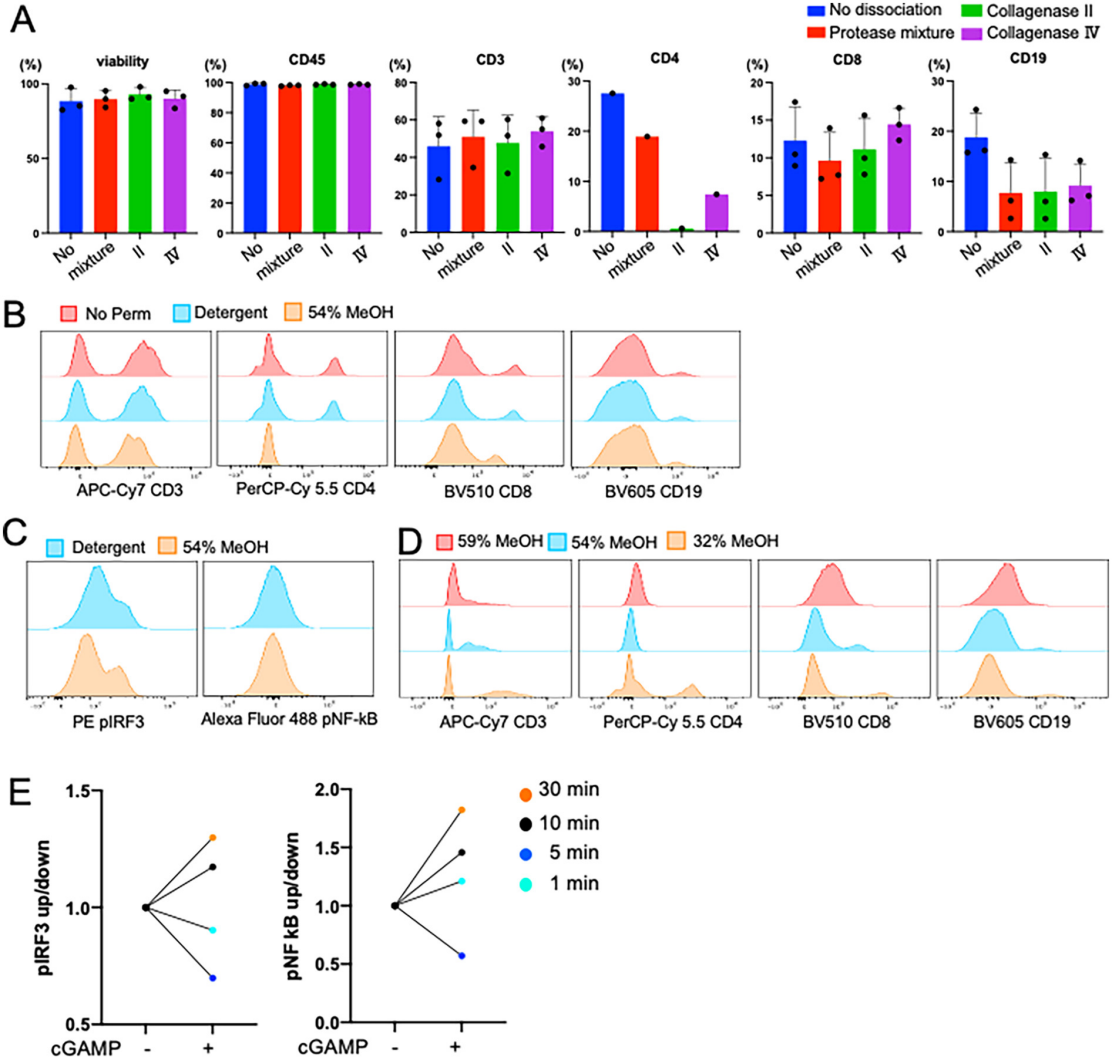
Dissociation proteases differentially affect surface epitopes. **(A)** Comparison of three dissociation methods using PBMC from the same matched participant. Collagenase Ⅳ showed the highest viability and cell surface marker frequencies. Viability was measured by live dead UV blue staining kit (Invitrogen). While CD45, CD3, and CD8 epitopes are less affected by dissociation, CD4 shows differential epitope cleavage with commercial protease mixture showing less cleavage than IV or II. Error bar means SD. Cell surface markers are percent of parents. **(B,C)** Comparison of detergent and MeOH permeabilization methods using PBMC of the same subject. Cells were permeabilized with 500 ml of each buffer for 30 min. 54% MeOH permeabilization reduced detection of the CD4 epitopes. **(D)** Optimization of the concentration of MeOH for permeabilization method. Cell surface markers were detectable in 32% of MeOH permeabilization. **(E)** Optimizing the time of permeabilization from 1 to 30 min after agonisms with 10 mg/ml of cGAMP stimulated PBMC. To fully permeabilize the cells, 30 min of incubation was required.

High-quality single-cell suspensions are essential for analyzing cell populations from biological specimens using flow cytometry and single cell RNA sequencing (scRNAseq). Achieving conditions that are optimal for both has clear benefits for investigators working with small tissue specimens. We previously published conditions for the dissociation of MSG for scRNAseq (6), however these conditions yielded variable results in flow cytometry due in large part to epitope sensitivity to dissociation enzymes. Tissue dissociation requires the use of proteases to break down the extracellular matrix and disrupt cell-cell interactions to achieve a single cell suspension. However, the preservation of the epitopes recognized by flow cytometry antibodies is necessary for downstream analytics. To understand epitope preservation, we tested the sensitivity of epitopes to dissociation enzymes on cell surface markers. Using PBMCs from healthy donors, cells were “dissociated” using three methods: collagenase II, collagenase IV, or a commercially available gentle protease mixture (i.e., Multi Tissue Dissociation Kit 1) following standard conditions (4). No significant differences in cell viability or the detection of CD45, CD3, or CD8 epitopes were observed. However, dissociation variably compromised CD4 and CD19 epitopes (Figure 2A). CD4 epitopes were most sensitive to dissociation with collagenase II resulting in a near total absence of detectable CD4+ T cells, whereas collagenase IV and protease mixture lost 73% and 31% of the total detectable T cells, respectively (Figure 2A). Reduced detection of CD19 epitopes (48%–54%) was also observed in dissociated cells. Overall, collagenase IV dissociation yielded numerically higher detection of CD3+ and CD8+ T cells (Figure 2A), consistent with findings reported for oral mucosal tissues (19). Overall, both protease mixture and collagenase IV reliably preserved most immune cell surface epitopes during tissue dissociation. Notably, CD4+ epitopes are sensitive to dissociation conditions; careful consideration of cell-type calls, especially when identifying CD4+ T cell subsets, is required.

The performance and application of flow cytometry can be expanded when additionally used to measure the expression of intracellular proteins but requires permeabilizing cells to access intracellular epitopes (4, 20). Like tissue dissociation, the choice of permeabilization methodology can impact the preservation and detection of both surface and intracellular markers (21). To optimize permeabilization conditions, we tested various parameters—including the presence of detergent, methanol concentration, and incubation times—using PBMCs from healthy donors. Detergent-based permeabilization was found to be mild and preserved the detection of surface markers such as CD4, CD8, and CD19 (Figure 2B). In contrast, methanol-based permeabilization enabled improved the detection of intracellular phosphorylated proteins as shown by clear positive peaks and increased MFI in pIRF3 (Figure 2C). Further optimization of methanol conditions revealed that 32% methanol at −20°C for 30 min provided superior performance for intracellular protein detection demonstrating less damage of cell surface epitope and highest staining status of both intracellular markers (Figures 2D). To validate our intracellular staining for pathway activation, we stimulated PBMCs with 10 µg/ml 2′3′-cGAMP, a potent Type-I IFN inducer, and measured the phosphorylation status of IRF3 and NF-κB (Figures 2E). The results underscore the need to optimize permeabilization protocols for intracellular and surface marker analysis.

### Dissociation of Minor salivary glands

**Figure 3:**
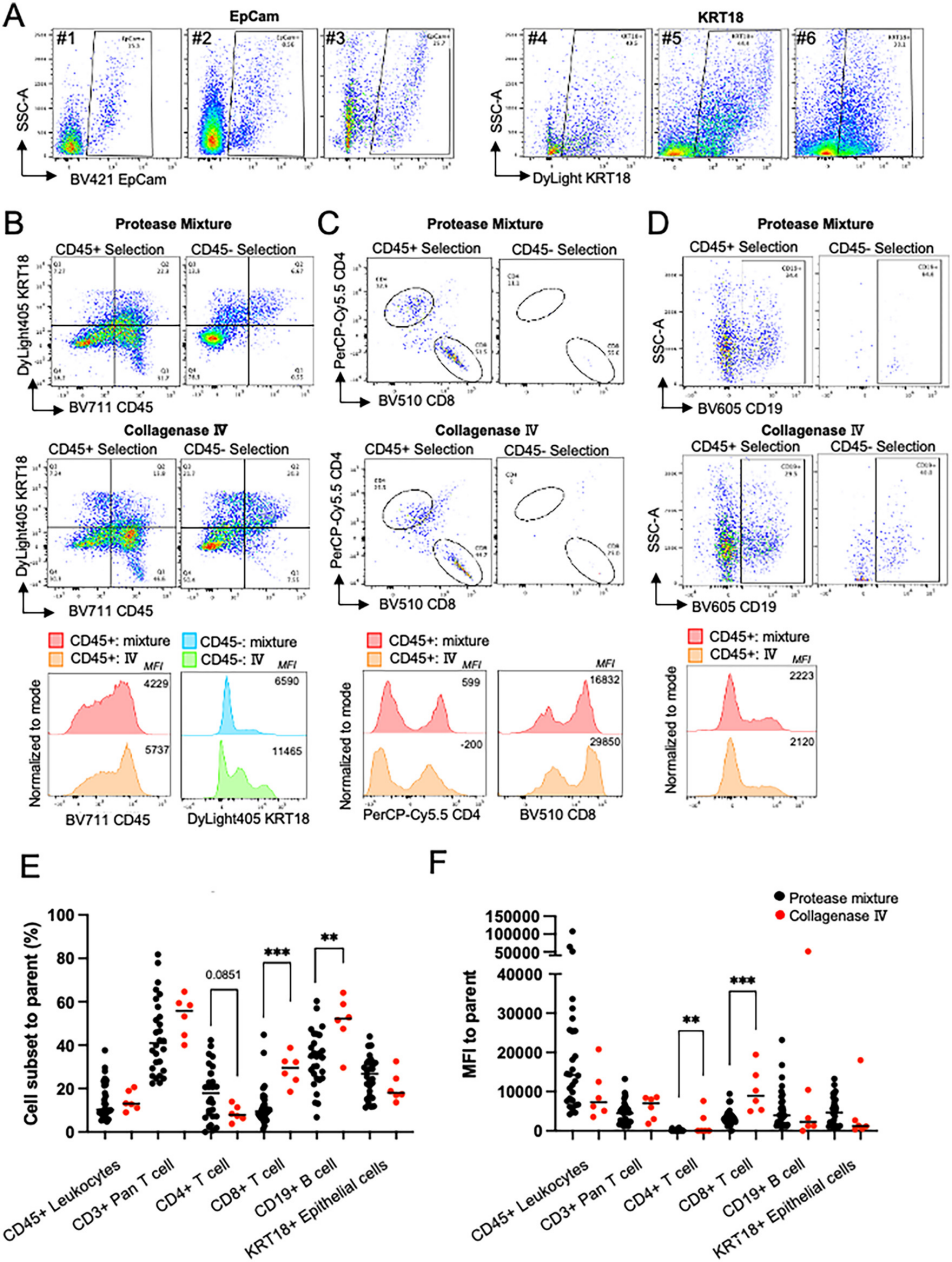
Dissociation optimization. **(A)** Comparison of fluorescence activated cell sorting plots (FACS) showing the distribution of cells between SSC-A and EpCam (Samples #1–3) and KRT18 (#4–6); KRT18 stained epithelial cells showed a higher frequency epithelial cells in MSG. *Plots gated singlets and cells and excluded debris.* **(B-D)** To compare protease mixture and collagenase IV to choose better method based on 1) cell surface markers and 2) lower nonspecific bindings, magnetically selected CD45+/- cells were used. The number in plot is %, and the number in histogram is mean fluorescence intensity (MFI). **(B)** Higher CD45+ and KRT18+ frequency and MFI in CD45+ and CD45- cells, respectively *Plots gated as single cells.* **(C)** Collagenase Ⅳ dissociated cells permitted higher CD8+ T cell frequency and MFI in CD45+ cells. *Plots are gated as CD45*+ *and CD3*+. **(D)** Both dissociation methos showed similar CD19 frequency and intensity*. Plots gated as singlets, cells, CD45+, and CD3-.* **(E,F)** Cell subset frequencies represented as percent (%) of parent fraction or MFI dissociated in protease mixture (black) or collagenase Ⅳ (red). Unpaired *t* test, ** = *p* < .01, *** = *p* < .001.

Next, we optimized MSG tissue preparation. To distinguish epithelial cells from lymphocytes, we initially tested EpCam (CD326) antibody as an epithelial cell marker. However, positive signal was observed in only 9%–26% (*n* = 3) of the single cells in suspension, far fewer than expected (∼50%, Figure 3A). EpCam is a surface marker that is plausibly sensitive to dissociation. We then tested KRT18, an intracellular epithelial marker. Unlike EpCam, KRT18 epitopes are intracellular and resistant to the dissociation conditions used. KRT18 positivity ranged from 30%–50% (*n* = 3) of the single cells in suspension (Figure 3A). Based on these findings we conclude that KRT18 is a more reliable marker for the detection of epithelial cells when digestion of tissue is required.

Next, we evaluated the effects of protease mixture and collagenase IV dissociation methods on MSG, focusing on cell viability, epitope preservation, and immune cell selection. Both methods maintained high cell viability (>90%). To assess their impact on epitope sensitivity and positive selection, a single patient's MSG biopsy was processed using either method, followed by CD45+ magnetic bead selection.

Flow cytometry analysis showed comparable CD45+ selection efficiency, with collagenase IV demonstrating a slight performance advantage (Figure 3B). Collagenase IV dissociation yielded higher frequencies of KRT18+ epithelial cells in the CD45- fraction and increased CD45+ immune cells in the CD45+ fraction (1.5-fold and 3.0-fold, respectively; Figure 3B). Mean fluorescence intensity (MFI) of CD45 and KRT18 was also higher, indicating better epitope preservation. In CD3+ T cells, collagenase IV resulted in slightly lower CD4+ and CD8+ T cell frequencies (0.78-fold and 0.88-fold compared to protease mixture) but increased CD8 MFI (Figure 3C). In the CD45- population, presumed to contain epithelial and stromal cells, collagenase IV reduced nonspecific CD4+ and CD8+ antibody binding. CD19+ B cell frequency and MFI were comparable between methods (Figure 3D).

Previously, we utilized the protease mixture for both flow cytometry and scRNAseq analysis of MSG, identifying pathological activation and viral entry factors across cell subpopulations (4–6). Comparing dissociation methods, collagenase IV (*n* = 6) increased CD8+ T cell frequency and significantly enhanced CD8+ MFI (*p* < 0.001; Figures 3E,F). Conversely, collagenase IV reduced CD4+ T cell frequency while increasing CD4+ MFI (*p* = 0.0041). The lower epithelial cell frequency observed with collagenase IV reflects a greater proportion of lymphocytes detected.

### Conventional flow cytometric analysis of human SjD MSG shows higher innate immune signaling

**Figure 4:**
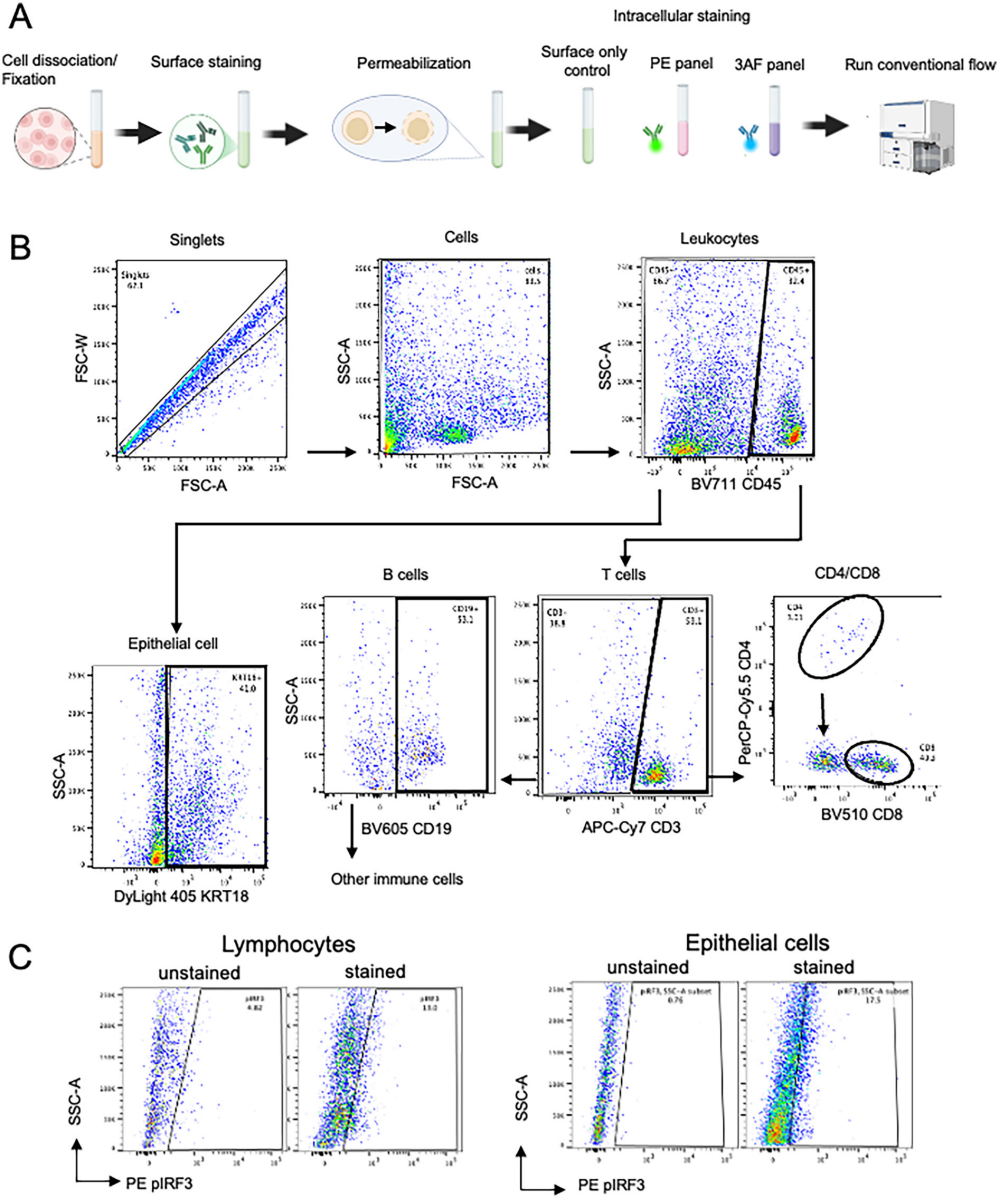
**(A)** Graphic overview of our optimized conventional flow panel. **(B,C)** Final gating strategy of salivary glands using the conventional panel for cell subset gating and intracellular markers.

A conventional flow cytometry panel was developed to detect epithelial cells, major immune cell subsets, and Type-I IFN pathway activation markers using a five-laser BD Symphony A5 cytometer with 27 fluorescent channels (Supplementary Table S3). After optimizing tissue dissociation, marker selection, permeabilization, and titration, we designed a panel to characterize MSG. Due to limitations in commercially available conjugated antibodies and instrument channel availability, single-cell suspensions were stained separately using PE and 3AF panels (Figure 4A). The final gating strategy for each cell subset is shown in Figure 4B. CD4+ T cell detection within CD3+ T cells (3.8%–14.0%) was lower than the expected 40%–60% for human T cells (22). Consequently, the low CD4+ T cell frequency precluded further gating for intracellular markers.

**Figure 5:**
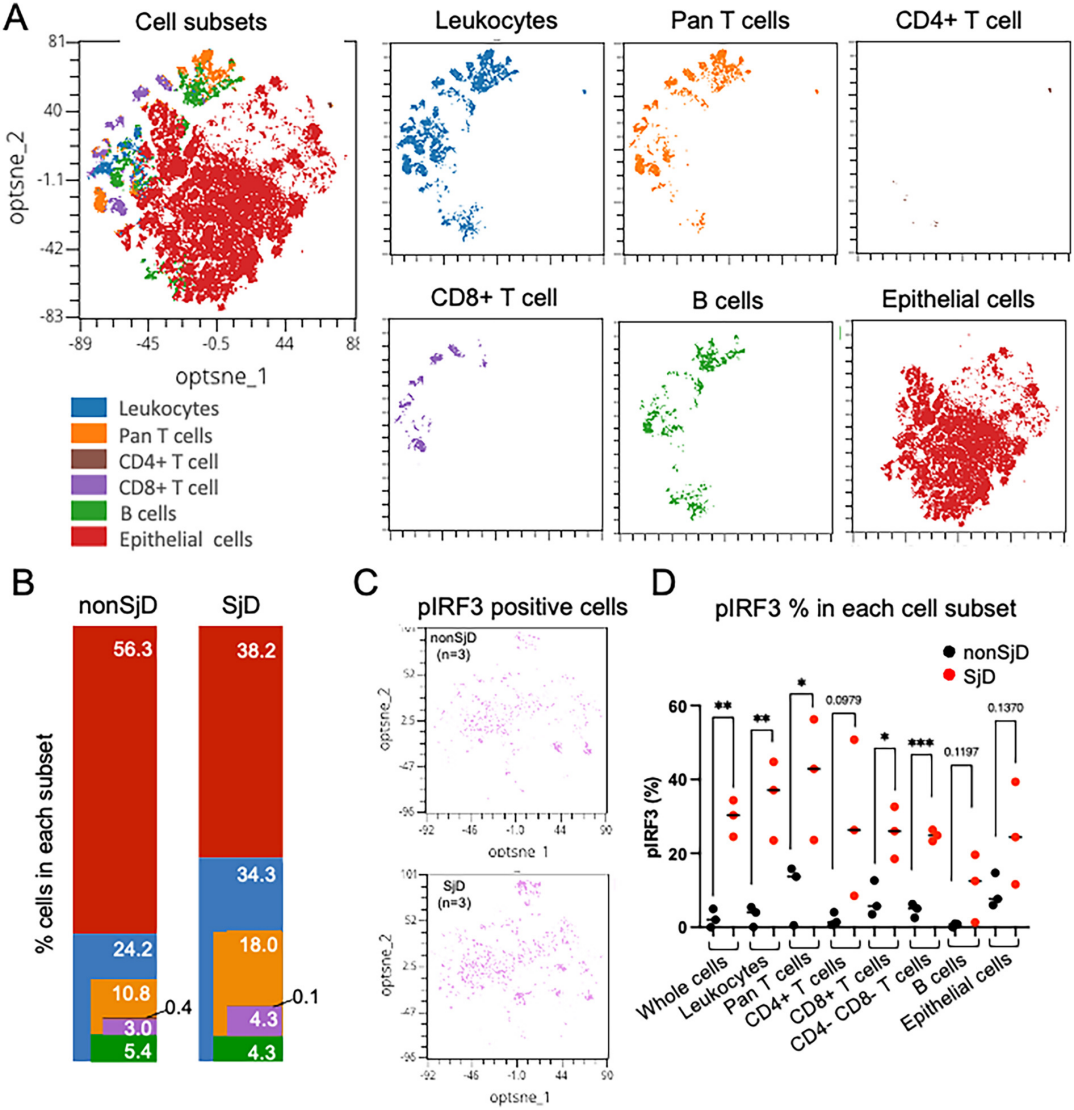
**(A)** Visualization of integrated high-dimensional flow cytometry data using optimized t-stochastic nearest neighbor embedding (opt-SNE) for exploration of clustering (OMIQ®). Individual flow studies were down-sampled to 8,000 singlet cells from each sample, clustered, and projected on to two dimensions (optsne_1, optnse_2). **(B)** Variability in cell frequencies, corresponding to colored clusters, are apparent between disease states (e.g., more T-lymphocytes in SjD). Color and order matched with cell distribution in *A*. **(C,D)** pIRF3 positive cells shown in opt-SNE and feature plot of pIRF3 positive cells in each group; SjD exhibits higher frequencies of positive signal in all cell subsets. Unpaired *t*-test, * = *p* < .05, ** = *p* < .01, *** = *p* < .001.

Intracellular staining of phosphorylated proteins was validated by comparing unstained and stained controls, confirming clear detection in both immune and epithelial cells (Figure 4C, Supplementary Figure S2). To demonstrate the capabilities of our conventional flow cytometry panel, we analyzed collagenase IV-dissociated MSG from SjD patients (*n* = 3) and non-SjD controls (*n* = 3). As expected, SjD MSG showed increased immune cell proportions, with CD45 + leukocytes and CD3+ T cells elevated 1.7-fold and 2.0-fold, respectively (Figures 5A,B).

Although based on small sample sizes, SjD samples showed higher frequencies of phosphorylated protein positive cells than controls, including an 11.0-fold (*p* = 0.0076) and 2.7-fold (*p* = 0.1370) increase in pIRF3 in leukocytes and epithelial cells, respectively (Figure 5C). Leukocytes also exhibited a 2.1-fold increase in pNF-κB (*p* = 0.0616), suggesting distinct activation dynamics across cell types. Notably, ∼50% of CD3+ T cells lacked CD4 or CD8 expression, likely due to CD4 epitope loss rather than true double-negative status. This “double-negative” population, enriched for Th cells, showed significantly elevated pIRF3 levels in SjD, consistent with trends in other subsets (Figure 5C). Additionally, a subset of leukocytes not classified as T or B cells likely included monocytes, dendritic cells, NK cells, and other immune populations.

### Frozen samples could be used as the same quality as fresh cells

**Figure 6:**
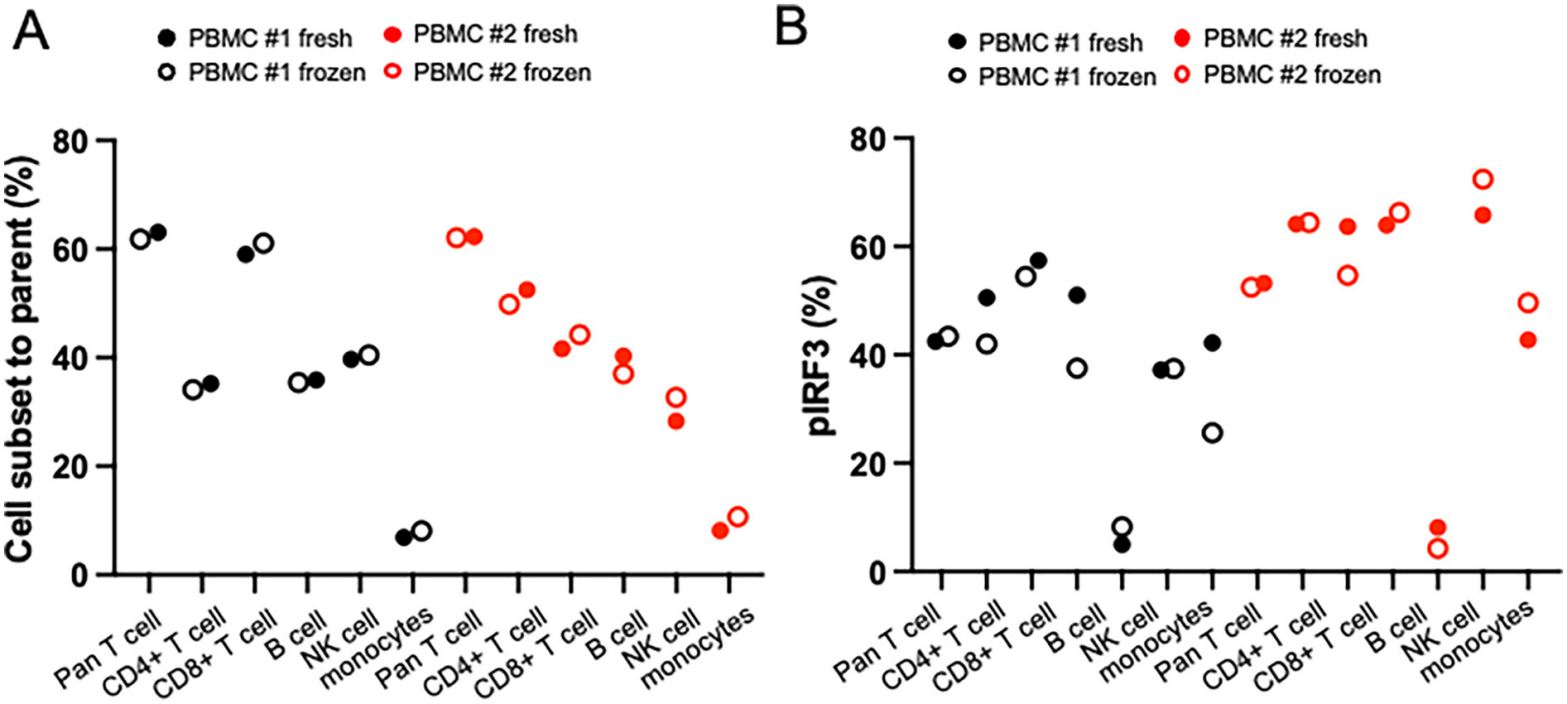
Comparison of fresh or frozen sample type using PBMCs from two different healthy donors. Matched fresh or frozen PBMC was stained and measured cell subset and phosphorylated protein level in different timing. **(A)** Cell subset frequencies showed nearly identical levels in fresh and frozen PBMCs in conventional methods. **(B)** Phosphorylated protein levels showed similar levels in fresh and frozen PBMCs in conventional and spectral methods.

We evaluated whether our conventional flow panel could be reliably applied to both fresh and viably cryopreserved samples by assessing immune cell frequencies and IRF3 phosphorylation in PBMCs from two donors with detectable pIRF3 levels. Cell frequencies were indistinguishable between fresh and cryopreserved samples, and pIRF3 expression remained highly similar (Figures 6A,B) without a discernible advantage for either condition. Although not directly tested, these findings support the use of cryopreserved MSG samples for our flow cytometry panels. Given proper cryopreservation (e.g., with CryoStor CS10), we expect immune cell frequencies and intracellular staining patterns to remain consistent with fresh samples.

## Discussion

Small, precious human tissue specimens (e.g., salivary gland biopsies) present both challenges and opportunities in translational research. Advances in single-cell technologies enable even limited samples to yield high-dimensional insights into complex cellular ecosystems. Flow cytometry remains a powerful tool for resolving immune and epithelial cell populations, with its greatest potential in complementing multimodal approaches such as single-cell RNA sequencing (scRNAseq). By generating single-cell suspensions, a fraction of the sample analyzed by flow cytometry can also be used for scRNAseq (∼10,000–20,000 cells), facilitating multiomic analyses (4, 5). Integrating cytometry with genomics extends beyond static histology, allowing for deeper interrogation of cell states, signaling pathways, and disease-specific perturbations.

We previously used a commercial protease mixture to dissociate MSG tissues for scRNAseq (4–6), while others have reported using collagenase I (23), TrypLE (24), or collagenase II (25). However, protease-mediated epitope cleavage led to poor alignment between scRNAseq and flow cytometry. To improve compatibility, we tested alternative dissociation methods and identified collagenase IV as the most reliable for preserving immune cell epitopes. While this approach resulted in mild cleavage of surface markers such as CD4 and CD19, it improved cell recovery and phenotypic resolution, similar to recent methods for immune cell isolation from oral mucosal tissues (19).

Having optimized tissue dissociation for flow cytometry compatibility, we next refined intracellular staining conditions to ensure robust detection of IFN pathway activation markers. IFN pathway markers such as pSTAT1 and pSTAT3 exhibit dynamic, disease-dependent localization (4). Methanol permeabilization provided superior intracellular staining and detection (26), though it can variably affect surface epitopes (27). Thus, careful optimization of antibody concentration and permeabilization duration is critical for reproducible phospho-flow analysis.

Using this optimized approach, we developed a high-parameter flow cytometry panel to immunophenotype MSG and assess Type-I IFN pathway activation. This panel identifies major immune and epithelial cell populations while detecting phosphorylation events indicative of immune activation. Notably, our data revealed lower-than-expected frequencies of CD4+ and CD8+ T cells in MSG, with a significant fraction of CD3+ T cells classified as double-negative (CD4^−^CD8^−^). This population was enriched in SjD and exhibited elevated pIRF3 levels, suggesting a functional relationship with Th cells. While double-negative T cells typically comprise 1%–3% of circulating lymphocytes in healthy blood (28) and are rare in MSG by scRNAseq (5). Our previous scRNAseq of MSG dissociated by protease mixture demonstrated CD4 to CD8 ratio around 0.7 (Supplementary Figure S4), suggesting this higher CD4^−^CD8^−^ population represents epitope-damaged CD4+ T cells. Alternative explanations include differences between 2D tissue sections and whole-organ dissociation approaches or potential contributions from other ungated immune cell populations. Future studies incorporating additional markers (e.g., D2–40, CD31, CD34) could help refine the characterization of these populations.

We measured cell viability using trypan blue immediately after tissue digestion to ensure that more than 90% cells were viable at fixation time and applied for staining. This method allows flexible staining timing on staining when is not possible to process sample immediately. However, adding fixable live dead staining would help to understand cell viability in each cell subset and apply only live cells for analysis if immediate staining after dissociation is possible.

Consistent with prior reports, we confirmed higher frequencies of infiltrating immune cells in SjD MSG (Figure 4B) (4). However, the relatively low immune cell infiltration in non-SjD controls limited direct comparisons of CD4+ T cell subsets. These findings underscore the importance of considering technical variables such as dissociation methodology and permeabilization effects when interpreting immune cell frequencies. Notably, pIRF3 levels were elevated across both leukocytes and epithelial cells in SjD, while pNF-κB phosphorylation was predominantly increased in leukocytes, suggesting differential activation of these pathways across cell types.

While previous studies employing flow cytometry in MSG focused primarily on leukocyte characterization with a limited set of 4–5 surface markers (29, 30), our approach expands on these efforts by integrating both surface and intracellular markers. Our high-parameter panel includes five surface markers for immune phenotyping and two intracellular markers (pIRF3 and pNF-κB) to assess IFN pathway activation. This design enables the simultaneous analysis of immune lineages, functional subsets, and activation states at the single-cell level. Furthermore, in the future the employment of spectral flow cytometer capable of detecting over 40 markers per run (31), our approach allows for greater flexibility in panel design and the inclusion of a broader range of phenotypic and functional markers.

Designed to capture key aspects of immune regulation, our panel enables a detailed interrogation of immune dynamics in MSG. By integrating surface and intracellular markers, it provides a scalable and reproducible framework for assessing cellular activation states at the single-cell level. When combined with multi-omic approaches like scRNAseq, phospho-proteomics, or spatial transcriptomics, this method enhances our ability to characterize immune responses in health and disease. Future studies in larger cohorts will be essential to validate these findings and further refine our understanding of SjD pathogenesis.

## Future directions

### Expanding marker panels to better define cell subsets

The salivary glands consist of acinar cells, ducts, stromal tissues (e.g., fibroblasts, endothelial cells), and tissue-resident and infiltrating immune cells. Fluid and protein secretion originate in acinar cells and are modified as they pass through the ducts to form saliva (8). To ensure representation of both epithelial and immune cell populations, our approach requires dividing MSG cell suspensions into three tubes: PE-panel, AF-panel, and a surface-stain-only control. This division reduces the total number of available cells for analysis and may impact gating resolution if suboptimal numbers of cells are used.

Differential interferon-stimulated gene expression and pathway activation in acinar and ductal cells have been reported in SjD (4). To refine epithelial subsetting, future iterations of our panel will incorporate acinar markers such as Aquaporin-5 or NKCC1, to distinguish acinar from ductal cells (32). Further stratification of acinar cells could include mucous and seromucous markers (e.g., Mucin-7, Mucin-5B), while Keratin-19 and Keratin-14 could help differentiate luminal and basal ductal cells from myoepithelial populations, andWFDC2 and SLC5A5 could be used to discriminate intercalated ducts from striated ducts, respectively (33). While highly interesting, this lies beyond the scope of our study.

The methodologies presented here provide a novel approach for flow cytometry in immunological studies of disease-affected MSG. While this study focused on conventional flow cytometry, we are confident that our panel can be adapted for spectral cytometry and will yield comparable results. This expands the potential of using MSG-derived single-cell suspensions for additional multiomic analyses (e.g., scRNAseq). Beyond salivary glands, our approach provides a framework for optimizing single-cell analyses in other tissues requiring dissociation (e.g., skin, lung, liver).

### Use of cryopreserved glands

To minimize batch effects, running all samples on the same day is ideal. While our study used only freshly biopsied MSG, prior research suggests minimal differences in immune phenotyping between fresh and cryopreserved PBMCs (34, 35). We confirmed that fresh and cryopreserved PBMCs show comparable immune cell distributions and IFN pathway activation (Figure 6). Additionally, our dissociation method applied to scRNAseq of cryopreserved MSG showed no unexpected shifts in cell proportions or IFN signaling activity (4). While cryopreserved MSG cells appear feasible for flow cytometry, validation is necessary before routine use.

### Limitations of flow cytometry when using minor salivary glands

#### Cell number constraints

Due to the limited tissue volume from a single patient biopsy, MSG cell yields range from 50,000–500,000 after dissociation, with potential losses during washing and staining. In our pilot studies, 30,000–50,000 cells were used per panel, a significantly lower number than required for PBMC analysis (typically ≥500,000 cells per sample) (36). Despite this, our conventional flow panel reliably detected most cell subsets and intracellular markers with an average of 35,000 cells per tube.

#### Compensation challenges

Accurate compensation is critical for reliable multiparameter flow cytometry, as errors can lead to misinterpretation. Ideally, the same cell types should be used for compensation, but when cell availability is limited or signals are weak, compensation beads serve as an alternative. However, certain commercial beads require additional validation before use (37). Given the limited number of MSG cells, we used PBMCs for compensation, as immune cells in MSG originate from blood, and HSG cell lines for KRT18+ epithelial markers. However, KRT18+ epithelial marker calibration requires further optimization (37).

## Summary

The panels and methodologies outlined here provide a framework for using MSG-derived cells in flow cytometry for cross-sectional and longitudinal immune studies. Our conventional flow panel captures key immune populations and activation markers, making it applicable even with limited cell numbers. For the first time, we demonstrate that flow cytometry can directly mark phosphorylation of interferon regulatory factors (i.e., IRF3) directly upstream of Type-I IFN signaling in putative target cells (e.g., secretory epithelial cells) the salivary glands of SjD patients. The ability to integrate such analyses into small tissue biopsies could enhance biomarker discovery, disease classification, and personalized treatment strategies—bringing us closer to defining the cellular and molecular signatures of human disease at the single-cell level.

## Data Availability

The datasets presented in this study can be found in online repositories. The names of the repository/repositories and accession number(s) can be found in the article/Supplementary Material.
