# Supplementary material for: Multiparameter flow cytometry enables immune profiling and IFN pathway analysis in human minor salivary glands
Source: Front Dent Med. 2025 Jun 12;6:1590516. doi: 10.3389/fdmed.2025.1590516 (PMC12198183; doi:10.3389/fdmed.2025.1590516)
Supplement: Supplementary file 1 [file Presentation1.pptx]

## Slide 1
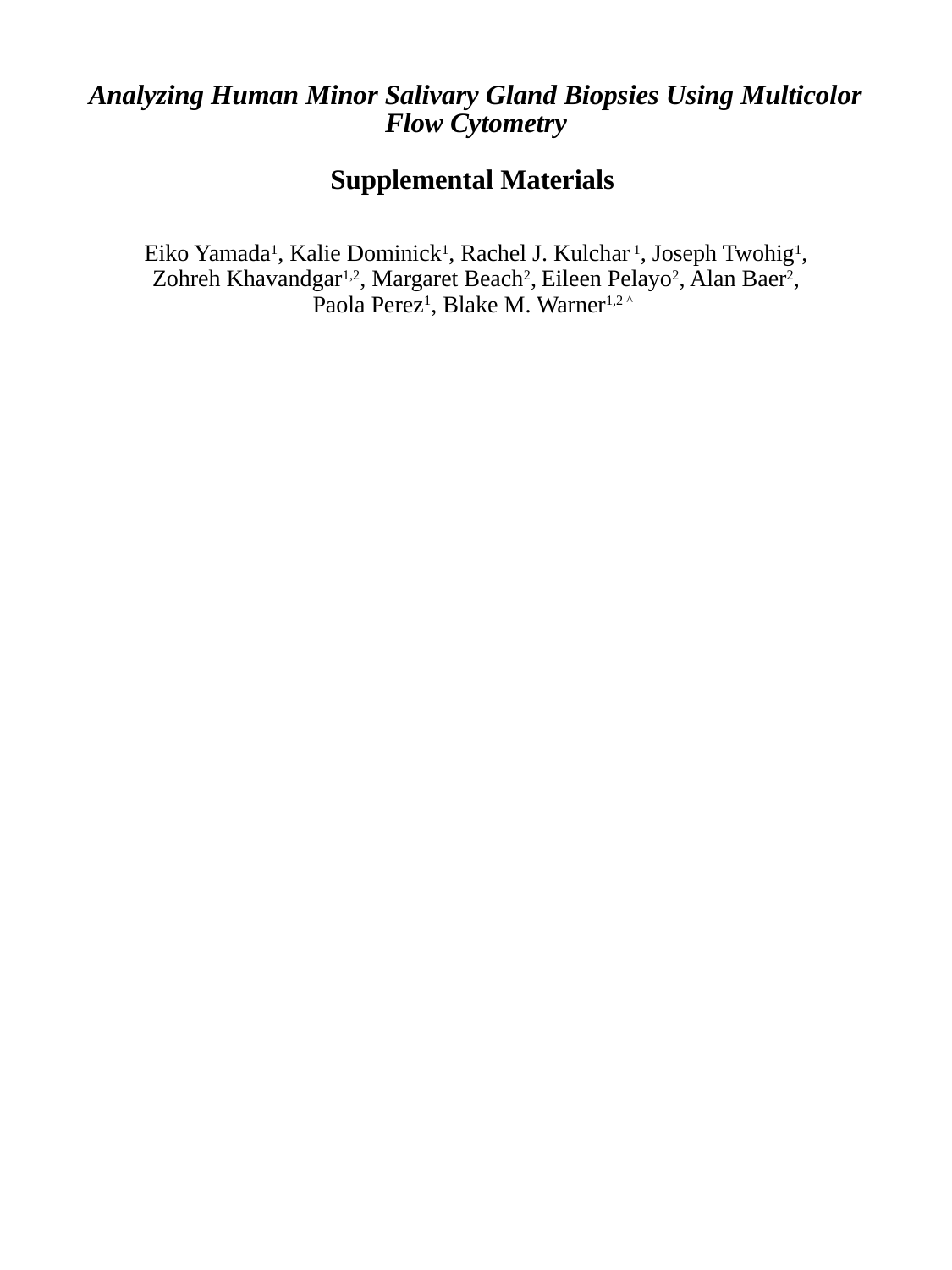

# Analyzing Human Minor Salivary Gland Biopsies Using Multicolor Flow Cytometry Supplemental Materials
Eiko Yamada1, Kalie Dominick1, Rachel J. Kulchar 1, Joseph Twohig1, Zohreh Khavandgar1,2, Margaret Beach2, Eileen Pelayo2, Alan Baer2, Paola Perez1, Blake M. Warner1,2 ^

## Slide 2
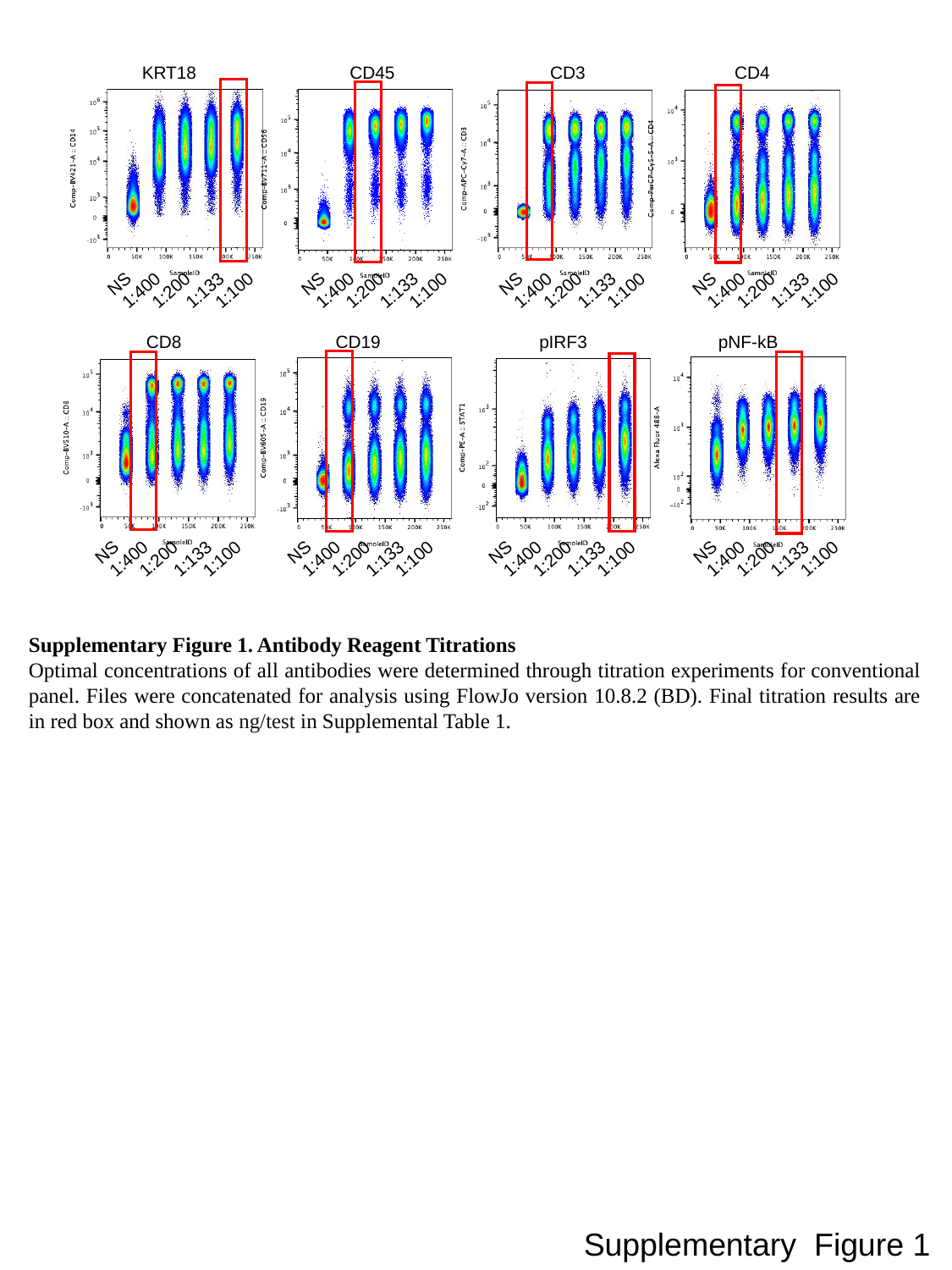

CD3
CD4
KRT18
CD45
NS
NS
NS
NS
1:400
1:400
1:400
1:400
1:133
1:133
1:133
1:133
1:100
1:100
1:100
1:100
1:200
1:200
1:200
1:200
CD8
CD19
pIRF3
pNF-kB
NS
NS
NS
NS
1:400
1:400
1:400
1:400
1:133
1:133
1:133
1:133
1:100
1:100
1:100
1:100
1:200
1:200
1:200
1:200
Supplementary Figure 1. Antibody Reagent Titrations
Optimal concentrations of all antibodies were determined through titration experiments for conventional panel. Files were concatenated for analysis using FlowJo version 10.8.2 (BD). Final titration results are in red box and shown as ng/test in Supplemental Table 1.
Supplementary Figure 1

## Slide 3
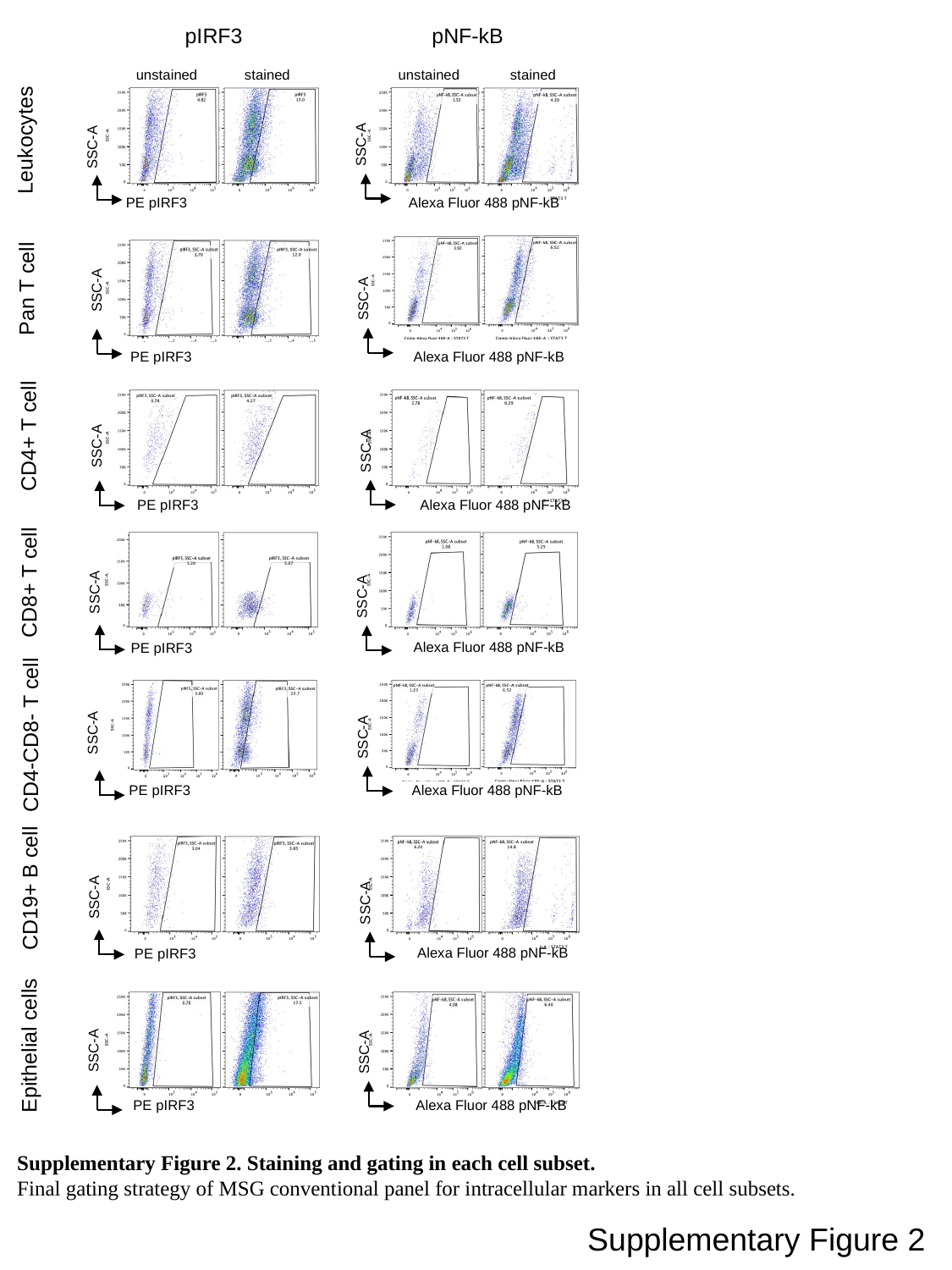

pIRF3
pNF-kB
unstained
stained
unstained
stained
Leukocytes
SSC-A
SSC-A
 Alexa Fluor 488 pNF-kB
PE pIRF3
Pan T cell
SSC-A
SSC-A
 Alexa Fluor 488 pNF-kB
PE pIRF3
CD4+ T cell
SSC-A
SSC-A
 Alexa Fluor 488 pNF-kB
PE pIRF3
CD8+ T cell
SSC-A
SSC-A
 Alexa Fluor 488 pNF-kB
PE pIRF3
CD4-CD8- T cell
SSC-A
SSC-A
PE pIRF3
 Alexa Fluor 488 pNF-kB
CD19+ B cell
SSC-A
SSC-A
 Alexa Fluor 488 pNF-kB
PE pIRF3
Epithelial cells
SSC-A
SSC-A
 Alexa Fluor 488 pNF-kB
PE pIRF3
Supplementary Figure 2. Staining and gating in each cell subset.
Final gating strategy of MSG conventional panel for intracellular markers in all cell subsets.
Supplementary Figure 2

## Slide 4
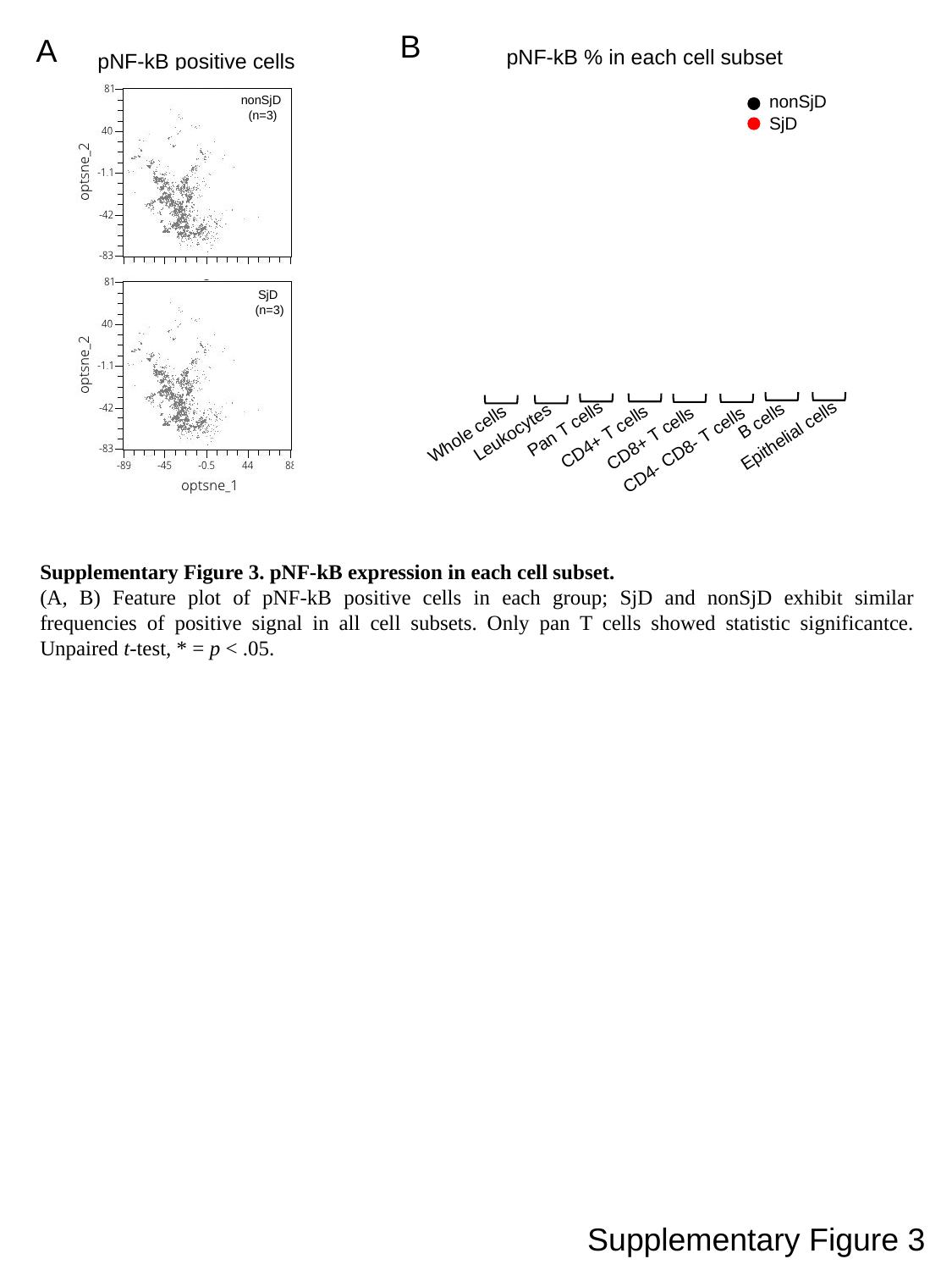

B
A
pNF-kB % in each cell subset
pNF-kB positive cells
nonSjD
nonSjD
(n=3)
SjD
SjD
(n=3)
B cells
Pan T cells
CD8+ T cells
Leukocytes
Whole cells
CD4+ T cells
CD4- CD8- T cells
Epithelial cells
Supplementary Figure 3. pNF-kB expression in each cell subset.
(A, B) Feature plot of pNF-kB positive cells in each group; SjD and nonSjD exhibit similar frequencies of positive signal in all cell subsets. Only pan T cells showed statistic significantce. Unpaired t-test, * = p < .05.
Supplementary Figure 3

## Slide 5
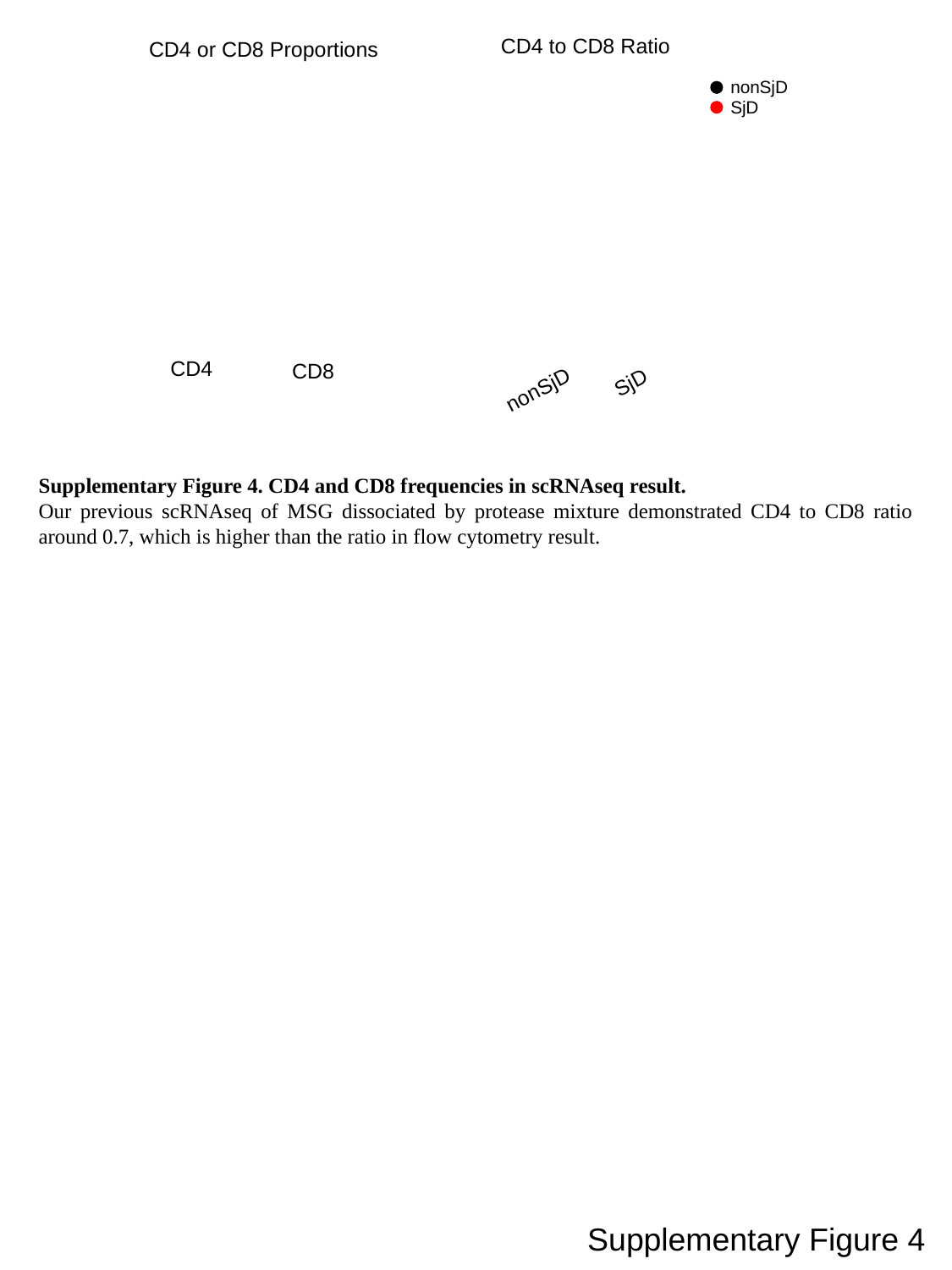

CD4 to CD8 Ratio
CD4 or CD8 Proportions
nonSjD
SjD
CD4
CD8
SjD
nonSjD
Supplementary Figure 4. CD4 and CD8 frequencies in scRNAseq result.
Our previous scRNAseq of MSG dissociated by protease mixture demonstrated CD4 to CD8 ratio around 0.7, which is higher than the ratio in flow cytometry result.
Supplementary Figure 4

## Slide 6
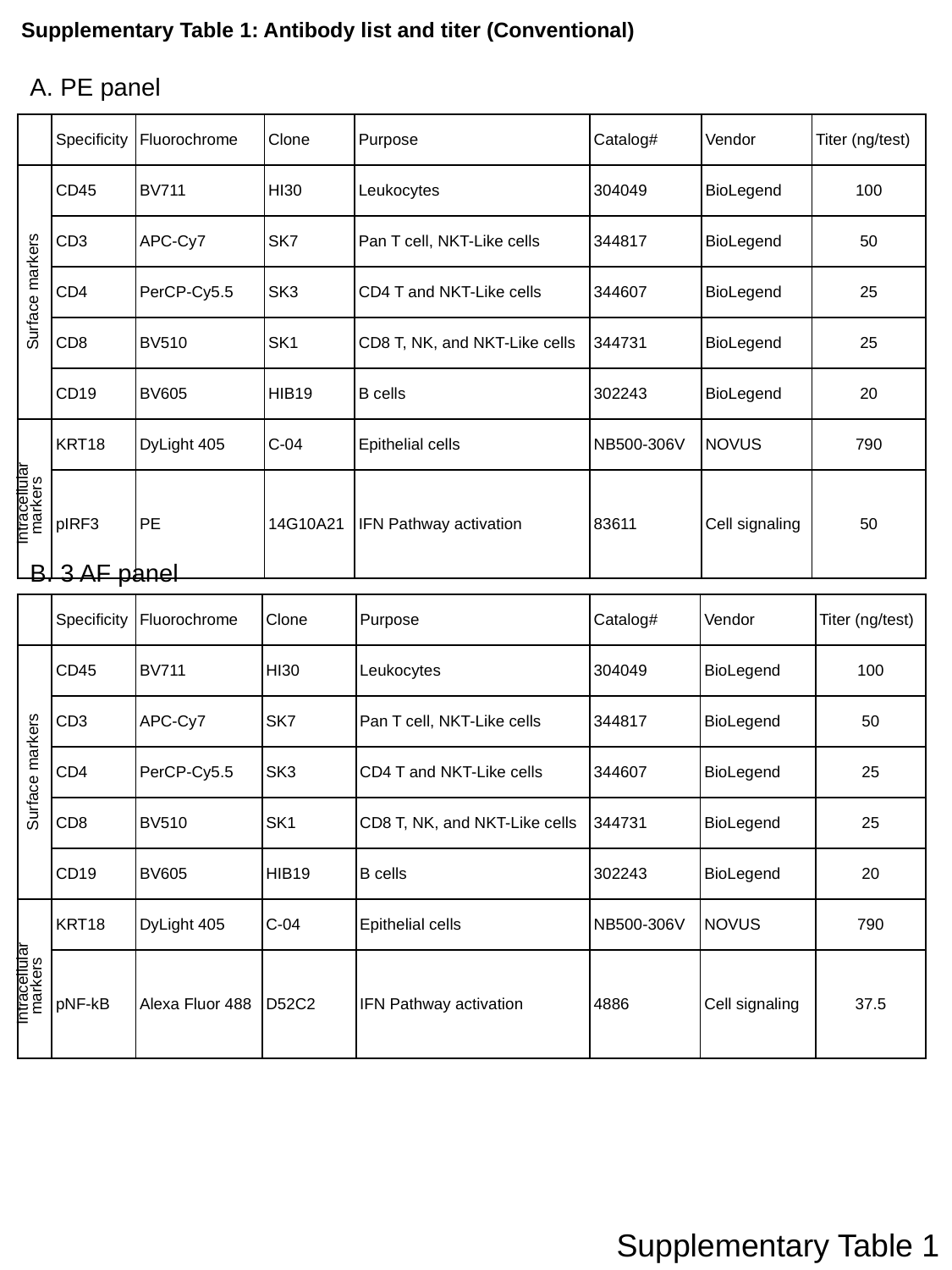

Supplementary Table 1: Antibody list and titer (Conventional)
A. PE panel
| | Specificity | Fluorochrome | Clone | Purpose | Catalog# | Vendor | Titer (ng/test) |
| --- | --- | --- | --- | --- | --- | --- | --- |
| Surface markers | CD45 | BV711 | HI30 | Leukocytes | 304049 | BioLegend | 100 |
| | CD3 | APC-Cy7 | SK7 | Pan T cell, NKT-Like cells | 344817 | BioLegend | 50 |
| | CD4 | PerCP-Cy5.5 | SK3 | CD4 T and NKT-Like cells | 344607 | BioLegend | 25 |
| | CD8 | BV510 | SK1 | CD8 T, NK, and NKT-Like cells | 344731 | BioLegend | 25 |
| | CD19 | BV605 | HIB19 | B cells | 302243 | BioLegend | 20 |
| Intracellular markers | KRT18 | DyLight 405 | C-04 | Epithelial cells | NB500-306V | NOVUS | 790 |
| | pIRF3 | PE | 14G10A21 | IFN Pathway activation | 83611 | Cell signaling | 50 |
B. 3 AF panel
| | Specificity | Fluorochrome | Clone | Purpose | Catalog# | Vendor | Titer (ng/test) |
| --- | --- | --- | --- | --- | --- | --- | --- |
| Surface markers | CD45 | BV711 | HI30 | Leukocytes | 304049 | BioLegend | 100 |
| | CD3 | APC-Cy7 | SK7 | Pan T cell, NKT-Like cells | 344817 | BioLegend | 50 |
| | CD4 | PerCP-Cy5.5 | SK3 | CD4 T and NKT-Like cells | 344607 | BioLegend | 25 |
| | CD8 | BV510 | SK1 | CD8 T, NK, and NKT-Like cells | 344731 | BioLegend | 25 |
| | CD19 | BV605 | HIB19 | B cells | 302243 | BioLegend | 20 |
| Intracellular markers | KRT18 | DyLight 405 | C-04 | Epithelial cells | NB500-306V | NOVUS | 790 |
| | pNF-kB | Alexa Fluor 488 | D52C2 | IFN Pathway activation | 4886 | Cell signaling | 37.5 |
Supplementary Table 1

## Slide 7
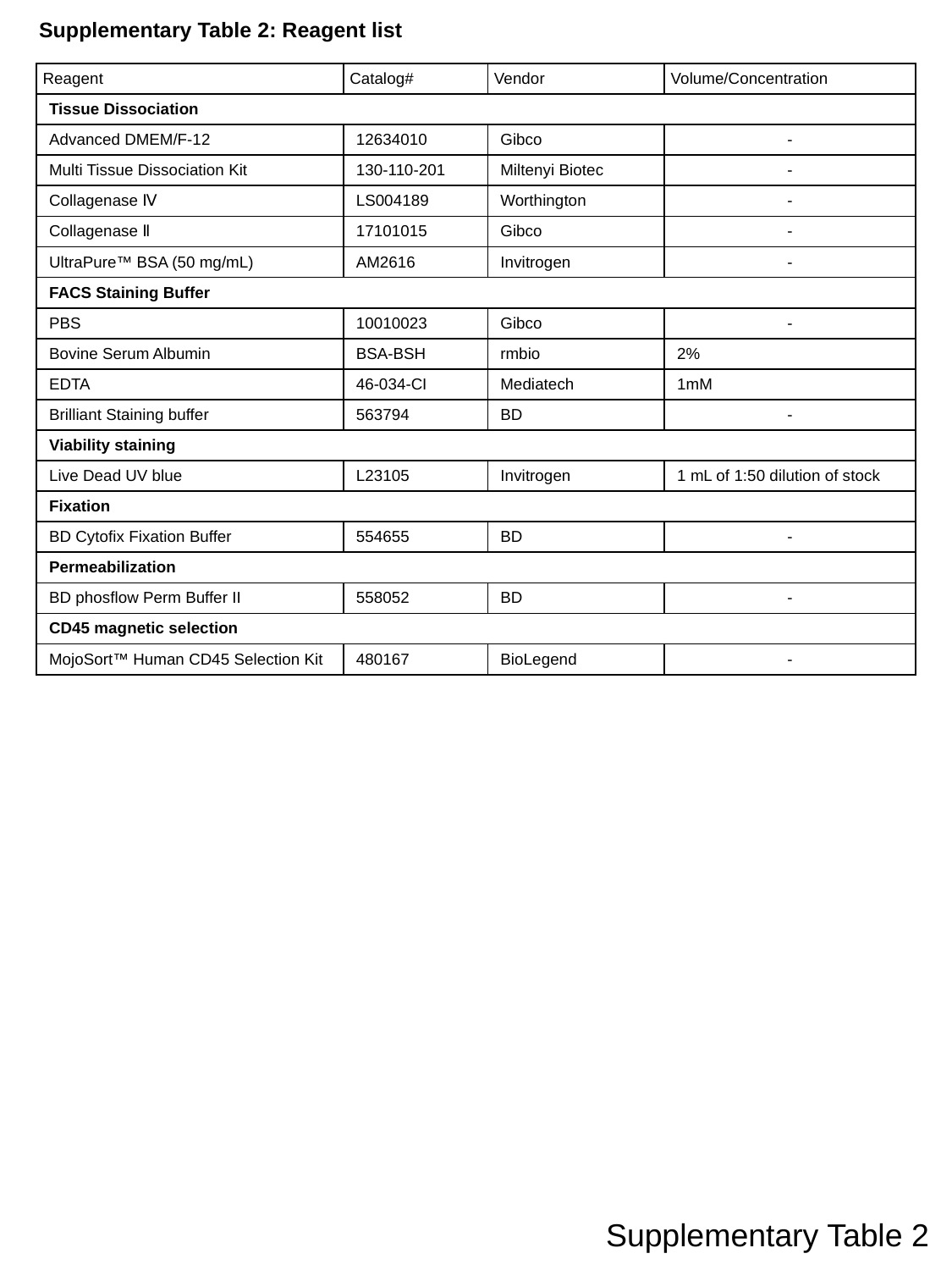

Supplementary Table 2: Reagent list
| Reagent | Catalog# | Vendor | Volume/Concentration |
| --- | --- | --- | --- |
| Tissue Dissociation | | | |
| Advanced DMEM/F-12 | 12634010 | Gibco | - |
| Multi Tissue Dissociation Kit | 130-110-201 | Miltenyi Biotec | - |
| Collagenase Ⅳ | LS004189 | Worthington | - |
| Collagenase Ⅱ | 17101015 | Gibco | - |
| UltraPure™ BSA (50 mg/mL) | AM2616 | Invitrogen | - |
| FACS Staining Buffer | | | |
| PBS | 10010023 | Gibco | - |
| Bovine Serum Albumin | BSA-BSH | rmbio | 2% |
| EDTA | 46-034-CI | Mediatech | 1mM |
| Brilliant Staining buffer | 563794 | BD | - |
| Viability staining | | | |
| Live Dead UV blue | L23105 | Invitrogen | 1 mL of 1:50 dilution of stock |
| Fixation | | | |
| BD Cytofix Fixation Buffer | 554655 | BD | - |
| Permeabilization | | | |
| BD phosflow Perm Buffer II | 558052 | BD | - |
| CD45 magnetic selection | | | |
| MojoSort™ Human CD45 Selection Kit | 480167 | BioLegend | - |
Supplementary Table 2

## Slide 8
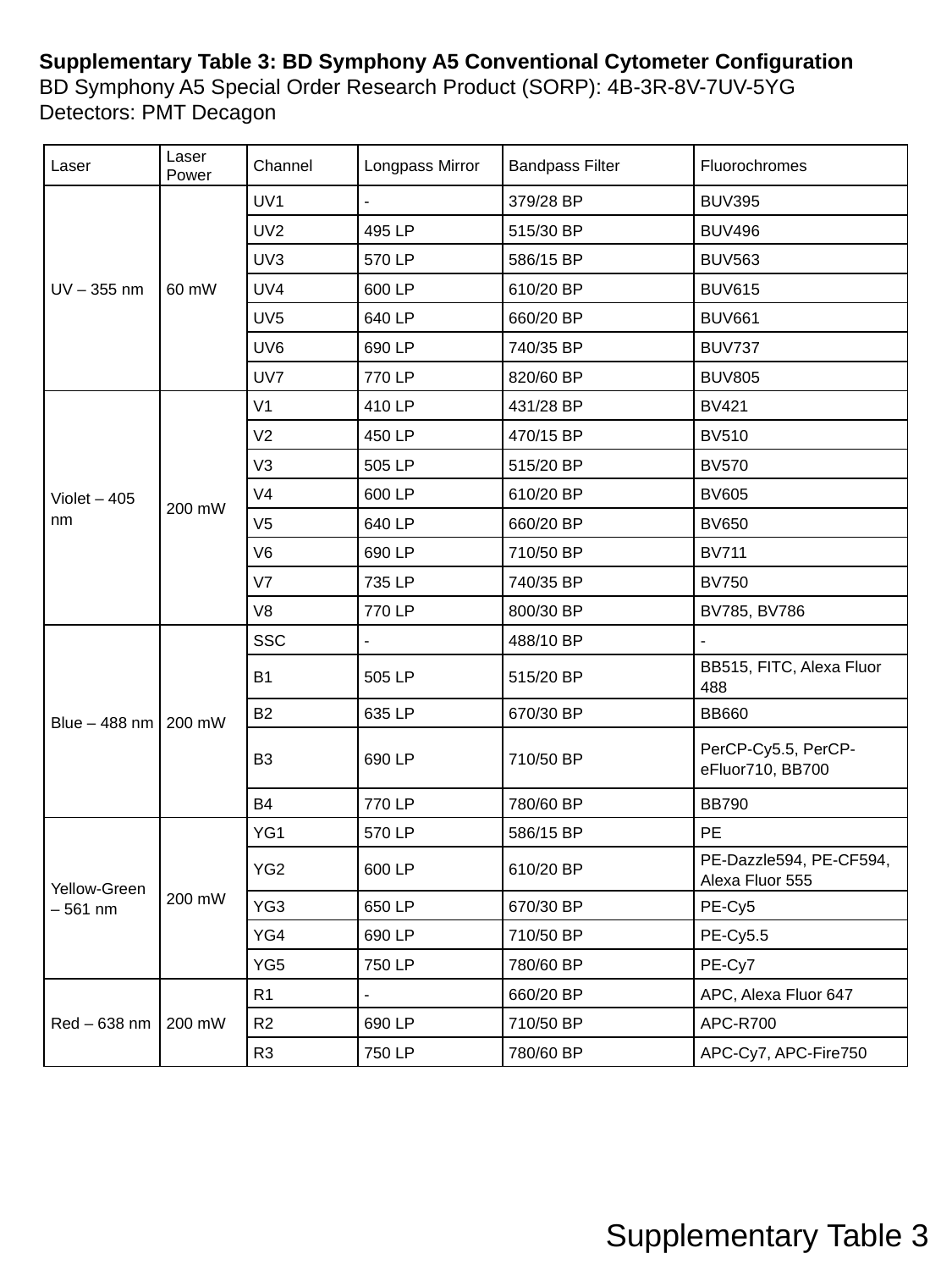

Supplementary Table 3: BD Symphony A5 Conventional Cytometer Configuration
BD Symphony A5 Special Order Research Product (SORP): 4B-3R-8V-7UV-5YG
Detectors: PMT Decagon
| Laser | Laser Power | Channel | Longpass Mirror | Bandpass Filter | Fluorochromes |
| --- | --- | --- | --- | --- | --- |
| UV – 355 nm | 60 mW | UV1 | - | 379/28 BP | BUV395 |
| | | UV2 | 495 LP | 515/30 BP | BUV496 |
| | | UV3 | 570 LP | 586/15 BP | BUV563 |
| | | UV4 | 600 LP | 610/20 BP | BUV615 |
| | | UV5 | 640 LP | 660/20 BP | BUV661 |
| | | UV6 | 690 LP | 740/35 BP | BUV737 |
| | | UV7 | 770 LP | 820/60 BP | BUV805 |
| Violet – 405 nm | 200 mW | V1 | 410 LP | 431/28 BP | BV421 |
| | | V2 | 450 LP | 470/15 BP | BV510 |
| | | V3 | 505 LP | 515/20 BP | BV570 |
| | | V4 | 600 LP | 610/20 BP | BV605 |
| | | V5 | 640 LP | 660/20 BP | BV650 |
| | | V6 | 690 LP | 710/50 BP | BV711 |
| | | V7 | 735 LP | 740/35 BP | BV750 |
| | | V8 | 770 LP | 800/30 BP | BV785, BV786 |
| Blue – 488 nm | 200 mW | SSC | - | 488/10 BP | - |
| | | B1 | 505 LP | 515/20 BP | BB515, FITC, Alexa Fluor 488 |
| | | B2 | 635 LP | 670/30 BP | BB660 |
| | | B3 | 690 LP | 710/50 BP | PerCP-Cy5.5, PerCP-eFluor710, BB700 |
| | | B4 | 770 LP | 780/60 BP | BB790 |
| Yellow-Green – 561 nm | 200 mW | YG1 | 570 LP | 586/15 BP | PE |
| | | YG2 | 600 LP | 610/20 BP | PE-Dazzle594, PE-CF594, Alexa Fluor 555 |
| | | YG3 | 650 LP | 670/30 BP | PE-Cy5 |
| | | YG4 | 690 LP | 710/50 BP | PE-Cy5.5 |
| | | YG5 | 750 LP | 780/60 BP | PE-Cy7 |
| Red – 638 nm | 200 mW | R1 | - | 660/20 BP | APC, Alexa Fluor 647 |
| | | R2 | 690 LP | 710/50 BP | APC-R700 |
| | | R3 | 750 LP | 780/60 BP | APC-Cy7, APC-Fire750 |
Supplementary Table 3

## Slide 9
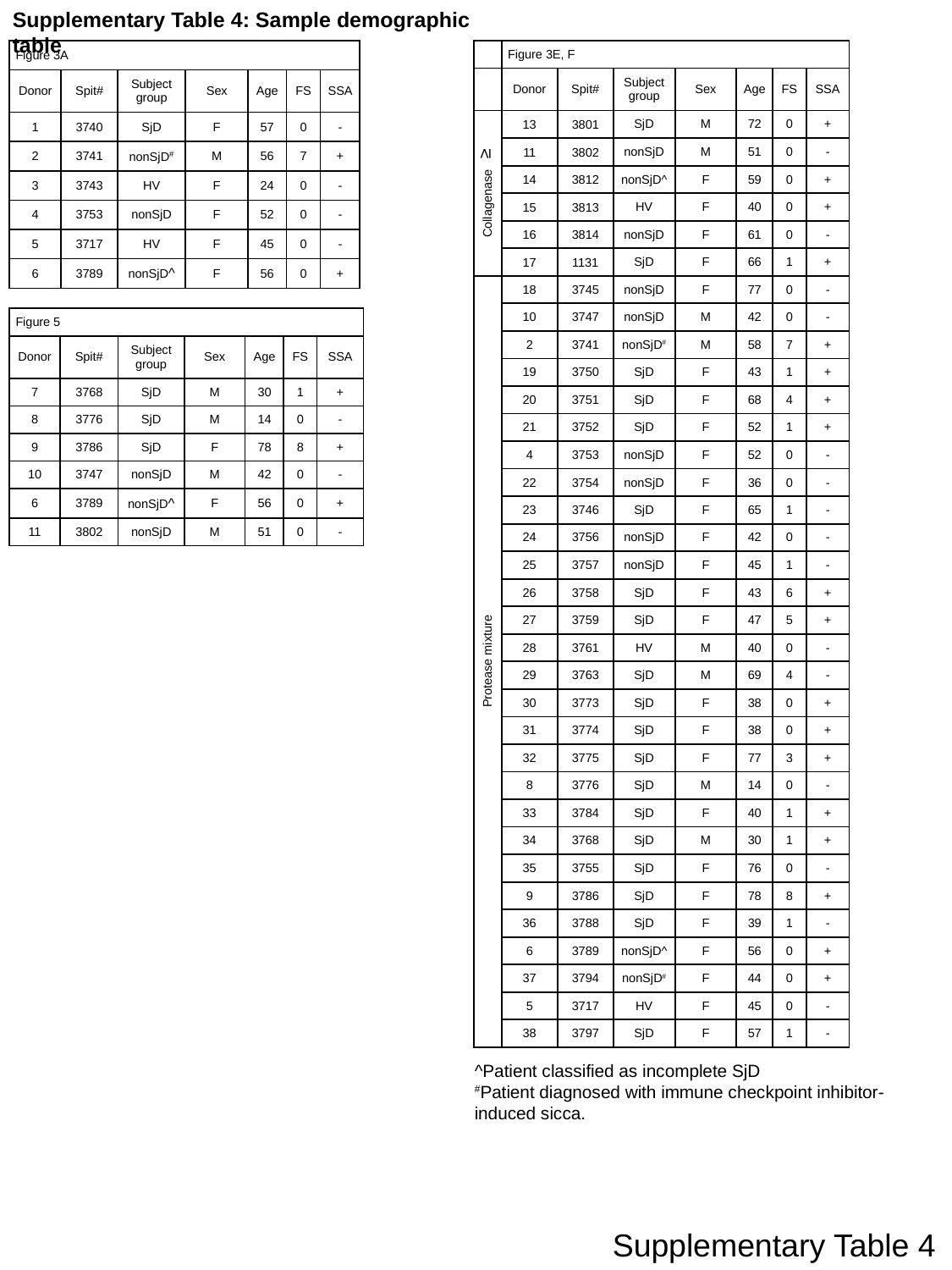

Supplementary Table 4: Sample demographic table
| Figure 3A | | | | | | |
| --- | --- | --- | --- | --- | --- | --- |
| Donor | Spit# | Subject group | Sex | Age | FS | SSA |
| 1 | 3740 | SjD | F | 57 | 0 | - |
| 2 | 3741 | nonSjD# | M | 56 | 7 | + |
| 3 | 3743 | HV | F | 24 | 0 | - |
| 4 | 3753 | nonSjD | F | 52 | 0 | - |
| 5 | 3717 | HV | F | 45 | 0 | - |
| 6 | 3789 | nonSjD^ | F | 56 | 0 | + |
| | Figure 3E, F | | | | | | |
| --- | --- | --- | --- | --- | --- | --- | --- |
| | Donor | Spit# | Subject group | Sex | Age | FS | SSA |
| Collagenase Ⅳ | 13 | 3801 | SjD | M | 72 | 0 | + |
| | 11 | 3802 | nonSjD | M | 51 | 0 | - |
| | 14 | 3812 | nonSjD^ | F | 59 | 0 | + |
| | 15 | 3813 | HV | F | 40 | 0 | + |
| | 16 | 3814 | nonSjD | F | 61 | 0 | - |
| | 17 | 1131 | SjD | F | 66 | 1 | + |
| Protease mixture | 18 | 3745 | nonSjD | F | 77 | 0 | - |
| | 10 | 3747 | nonSjD | M | 42 | 0 | - |
| | 2 | 3741 | nonSjD# | M | 58 | 7 | + |
| | 19 | 3750 | SjD | F | 43 | 1 | + |
| | 20 | 3751 | SjD | F | 68 | 4 | + |
| | 21 | 3752 | SjD | F | 52 | 1 | + |
| | 4 | 3753 | nonSjD | F | 52 | 0 | - |
| | 22 | 3754 | nonSjD | F | 36 | 0 | - |
| | 23 | 3746 | SjD | F | 65 | 1 | - |
| | 24 | 3756 | nonSjD | F | 42 | 0 | - |
| | 25 | 3757 | nonSjD | F | 45 | 1 | - |
| | 26 | 3758 | SjD | F | 43 | 6 | + |
| | 27 | 3759 | SjD | F | 47 | 5 | + |
| | 28 | 3761 | HV | M | 40 | 0 | - |
| | 29 | 3763 | SjD | M | 69 | 4 | - |
| | 30 | 3773 | SjD | F | 38 | 0 | + |
| | 31 | 3774 | SjD | F | 38 | 0 | + |
| | 32 | 3775 | SjD | F | 77 | 3 | + |
| | 8 | 3776 | SjD | M | 14 | 0 | - |
| | 33 | 3784 | SjD | F | 40 | 1 | + |
| | 34 | 3768 | SjD | M | 30 | 1 | + |
| | 35 | 3755 | SjD | F | 76 | 0 | - |
| | 9 | 3786 | SjD | F | 78 | 8 | + |
| | 36 | 3788 | SjD | F | 39 | 1 | - |
| | 6 | 3789 | nonSjD^ | F | 56 | 0 | + |
| | 37 | 3794 | nonSjD# | F | 44 | 0 | + |
| | 5 | 3717 | HV | F | 45 | 0 | - |
| | 38 | 3797 | SjD | F | 57 | 1 | - |
| Figure 5 | | | | | | |
| --- | --- | --- | --- | --- | --- | --- |
| Donor | Spit# | Subject group | Sex | Age | FS | SSA |
| 7 | 3768 | SjD | M | 30 | 1 | + |
| 8 | 3776 | SjD | M | 14 | 0 | - |
| 9 | 3786 | SjD | F | 78 | 8 | + |
| 10 | 3747 | nonSjD | M | 42 | 0 | - |
| 6 | 3789 | nonSjD^ | F | 56 | 0 | + |
| 11 | 3802 | nonSjD | M | 51 | 0 | - |
^Patient classified as incomplete SjD
#Patient diagnosed with immune checkpoint inhibitor-induced sicca.
Supplementary Table 4
